# First Evidence of the Effects of Polyethylene Terephthalate Microplastics on Ruminal Degradability and Gastro-Intestinal Digestibility of Mixed Hay

**DOI:** 10.3390/ani14152139

**Published:** 2024-07-23

**Authors:** Sonia Tassone, Salvatore Barbera, Hatsumi Kaihara, Sara Glorio Patrucco, Khalil Abid

**Affiliations:** Department of Agricultural, Forest and Food Sciences, University of Turin, Largo P. Braccini 2, 10095 Grugliasco, TO, Italy; sonia.tassone@unito.it (S.T.); salvatore.barbera@unito.it (S.B.); hatsumi.kaihara@unito.it (H.K.); sara.gloriopatrucco@unito.it (S.G.P.)

**Keywords:** microplastics, polyethylene terephthalate, ruminal degradability, gastro-intestinal digestibility, mixed hay

## Abstract

**Simple Summary:**

Simple Summary: Microplastics have emerged as a pressing global environmental issue and have been alarmingly detected in both the feed and feces of ruminants. However, their effects on the ruminal and intestinal digestive tract have not yet been studied. Our study represents the first investigation of the effects of polyethylene terephthalate microplastics on the ability of the ruminal-gastro-intestinal system to degrade and digest mixed hay. Our findings reveal that polyethylene terephthalate significantly reduce the digestibility of crude protein in mixed hay. Notably, low levels of PET MPs negatively impacted the intestinal phase, while medium and high levels disrupted the ruminal phase. Moreover, medium and high concentrations of polyethylene terephthalate hindered the degradation of fiber fractions, specifically of neutral detergent fiber. These insights underscore the potential risks posed by polyethylene terephthalate microplastics to ruminal–gastro-intestinal functionality and highlight the urgent need for innovative strategies to combat this emerging environmental challenge, ensuring the sustainability and productivity of ruminant farming.

**Abstract:**

Microplastics (MPs) raise environmental concerns. However, their effects on the ruminal–gastro-intestinal system have not yet been studied. This study aims to investigate the effects of polyethylene terephthalate (PET) MPs on the ability of the ruminal–gastro-intestinal system to degrade and digest mixed hay. Using a three-step in vitro ruminal–gastro-intestinal incubation system, PET MPs were introduced at concentrations of 0, 5, 10, and 15 g/L in ruminal and gastro-intestinal solutions. Ruminal fluid was collected from three 16-month-old Piedmontese bulls. The experiment was conducted on three mixed hays and was repeated three times, with triplicate incubations in each run. The results reveal that PET MPs reduced the degradability and digestibility of crude protein. Specifically, crude protein degradation was reduced by 9% at medium and 16% at high PET MP concentrations in the ruminal phase, while the crude protein digestibility of undegraded crude protein was reduced by 8% at the lowest PET MPs concentration in the gastro-intestinal tract. Additionally, PET MPs reduced the degradation of neutral detergent fiber at medium and high PET MP concentrations in the ruminal phase by 9% and 13%, respectively. These results highlight the risks of PET MPs contamination on ruminal–gastro-intestinal functions and underscore the urgent need to mitigate MPs contamination in the livestock sector.

## 1. Introduction

Microplastics (MPs), defined as plastic debris between 1 µm and 5 mm in size, have emerged as a global environmental problem affecting both marine and terrestrial ecosystems [1]. In terrestrial environments, the presence of MPs in soil contributes to the destruction of soil structure and microbial communities, which in turn leads to nutrient depletion and reduces plant growth and crop production [1]. When these MPs enter the bodies of animals and humans, they can cause physical harm, oxidative stress, cell death, cytotoxicity, neurotoxicity, reproductive toxicity, and immune system disruption [1,2]. Consequently, MPs have the potential to disrupt ecological processes, contributing to the population declines of certain species and altering community dynamics [1].

Recent studies have revealed alarming amounts of MPs in the digestive systems of wild prey birds, migratory birds, and ducks in intensive farming, with polyethylene terephthalate (PET) MPs and polyethylene MPs being the predominant MP polymers [3,4,5]. A recent pilot study conducted on an Italian dairy farm revealed that 100% of ryegrass hay samples were contaminated with MPs, averaging 39,300 ± 7020 MPs per kilogram of dry ryegrass hay [6]. Another study in India found that 100% of cow diet samples were contaminated with PET MPs, with alarming levels ranging from 89 to 326 g per kilogram of feed [7]. Additionally, a study in Spain reported the presence of MPs in 92% of sheep feces samples, with an average of 997 ± 971 particles per kilogram of dry feces [8]. These results confirm that PET MPs can be ingested by ruminants via feed and pass through the ruminal–gastro-intestinal tract.

Recent in vitro research, simulating the ruminal digestive system of cattle, found that about 1% of PET MPs can be degraded [9], releasing 0.15 mM terephthalic acid, mono-2-hydroxyethyl terephthalate, and bis(2-hydroxyethyl) terephthalate [10]. A study conducted on in vivo mouse models has shown that administering PET MPs at 4 mg/day over a short period of time (7 days) altered the intestinal microenvironment, changed the microbiota community, and reduced its diversity [11]. Additionally, oral administration of PET MPs at a rate of 3 × 10^4^ particles every 3 days over 8 weeks caused a slight chronological change in microbial composition [12]. For human, an in vitro study using a digestion model that simulated human colonic digestion, including fermentation of the gut microbiota, showed that ingestion of PET MPs at 166 mg/intake modified both the composition and diversity of the microbial communities in the human colon and decreased the amount of total viable bacteria [13]. Another in vitro study simulating human saliva and stomach and intestinal solutions demonstrated that adding PET MPs at 80 mg/L of small intestine solution reduced lipid digestion [14].

However, the effects of MPs evaluated on in vivo mouse models and in vitro models simulating the human digestive system cannot be fully extrapolated to ruminants, due to significant differences in their digestive systems [15]. Only non-toxic doses of MPs confirmed in mice can be applied to in vivo studies for ruminants, due to ethical restrictions [15]. Furthermore, the Three Rs (Replacement, Reduction, and Refinement) apply to animal studies, encouraging the development of alternative in vitro approaches to reduce the use of animals in research and replace them with simulated systems [16].

Given these considerations, our hypothesis is that PET MP contamination in the ruminal–gastro-intestinal system of bulls will adversely impact their digestive processes. Specifically, this study aims to investigate, for the first time, the effects of PET MP contamination in the ruminal–gastro-intestinal system of bulls on their ability to degrade and digest hay, using an in vitro simulated system.

## 2. Materials and Methods

### 2.1. Feedstuff Sample Preparation

In October 2023, first-cut mixed hay samples, one of the most crucial feed resources for ruminants globally [17], were systematically collected from three farms across the north region of Italy in the Piedmont region. The mixed hays included grasses (ryegrass, fescue, and timothy) and legumes (clover). Approximately 2 kg of mixed hay was collected from each farm, gathered from various round bales stored in different positions, to ensure a representative sampling of the hay used in these studies related to ruminant nutrition. These representative samples of mixed hay were then transported to the laboratory. 

The samples were dried at 60 °C for 24 h, ground to a particle size of 1 mm using a granulator (MLI 204; Bühler AG, Uzwil, Switzerland), and stored in glass bottles until further use. The chemical composition of the ground mixed hay was analyzed for dry matter (DM, #930.15), ash (#942.05), crude protein (CP, #984.13), and ether extract (EE, #942.05), according to AOAC methods [18]. Fiber fractions, including neutral detergent fiber (NDF), acid detergent fiber (ADF), and acid detergent lignin (ADL), were determined using an Ankom 200 Fiber Analyzer (Ankom Technology, Macedon, NY, USA) according to the procedures of Van Soest et al. [19]. The non-fiber carbohydrates (NFC) were calculated following the equation of NRC [20]:

(1) NFC=1000−CP−EE−NDF−ash

where

▪ NFC are non-fiber carbohydrates in mg/g dry matter;
▪ CP is crude protein in mg/g dry matter;
▪ EE is ether extract in mg/g dry matter;
▪ NDF is neutral detergent fiber in mg/g dry matter;
▪ Ash refers to ash in mg/g dry matter.

The chemical composition of the mixed hays used in this study is presented in Table 1.

The dried and ground mixed hay samples were weighed into Ankom filter bags (F57 filter bags with 25 μm porosity, Ankom Technology, Macedon, NY, USA) at 0.50 ± 0.01 g. In total, 108 bags containing mixed hays were prepared (36 bags/mixed hay), to be used in three different in vitro incubations.

### 2.2. Microplastic Characteristics

**Figure 1:**
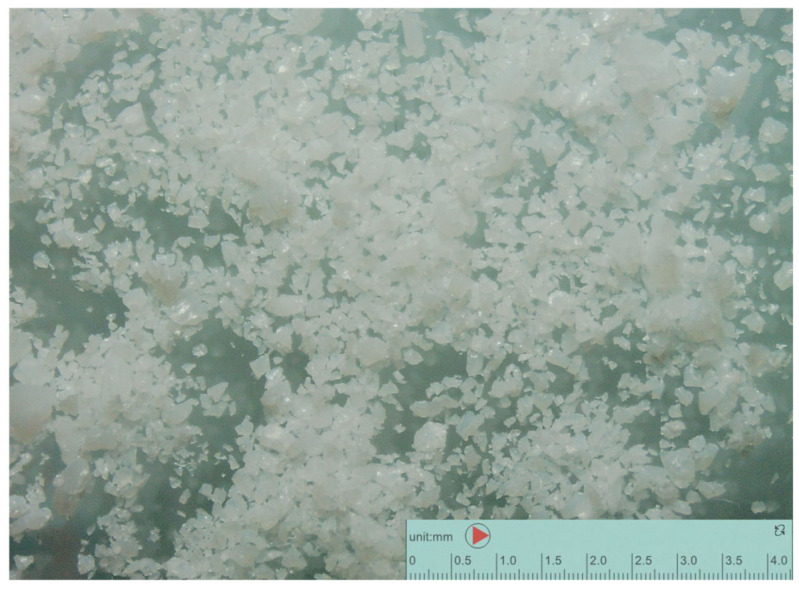
Stereomicroscope pictures of polyethylene terephthalate microplastics used in experiment.

**Figure 2:**
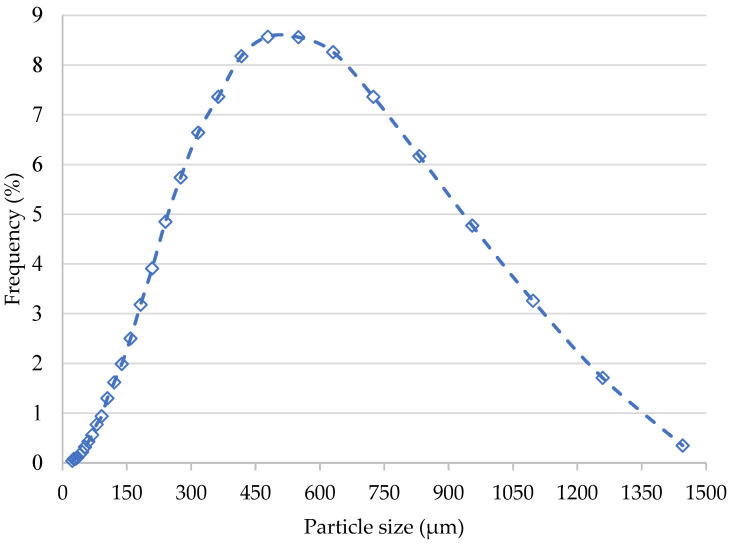
Distribution of the polyethylene terephthalate microplastic particle sizes used in the experiment, based on particle counts.

Pure PET MPs in granular form were provided from an Italian company and utilized without any additional processing that could alter their crystallinity or chemical reactivity. The PET MPs used were opaque and had a heterogeneous shape, as depicted in Figure 1, captured with a stereomicroscope (Nikon H550S, Nikon Corporation, Tokyo, Japan). These PET MPs have a density of 1.38 g/cm^3^ and an average diameter of 522 μm. The size distribution of the PET MPs is illustrated in Figure 2, based on data provided by the producer company.

### 2.3. In Vitro Ruminal Degradability and Gastro-Intestinal Digestibility Analysis

The experiment was conducted using simulated ruminal–gastro-intestinal digestion systems to investigate the effects of PET MPs on mixed hay ruminal degradability and gastro-intestinal digestibility, using a three-steps method [21,22]. The study included four treatment groups with varying concentrations of PET MPs: 0 g/L (control), 5 g/L, 10 g/L, and 15 g/L. This range of PET MP concentrations was chosen to investigate the dose-dependent effects on ruminal degradation and gastro-intestinal digestion. This study serves as the pioneering effort to explore the direct effects of MP contamination in this specific environment.

*Step* *1.*  *Ruminal degradation*

Ruminal degradation was performed using an Ankom Daisy^II^ incubator (Ankom Technology Corp., Fairport, NY, USA) and was repeated three times over three successive weeks (runs) in December 2023. Each run included three repetitions of mixed hay samples per jar. For each experimental run, 2 L of ruminal fluid was collected at a slaughterhouse, immediately after slaughter, from different positions within the rumen of three healthy Piedmontese bulls, following the standardized protocol of Fortina et al. [23]. The bulls were each approximately 16 months old and received a daily diet composed of 2 kg of barley straw, 4 kg of ryegrass hay, and 10 kg of concentrate. The concentrate was composed of 68.9% corn meal, 11.7% soybean meal, 5.9% beet pulp, 5% bran, 1% soybean oil, 1% extruded flaxseeds, 0.5% hydrogenated fat, 3.5% calcium carbonate, 0.5% tampon, 0.9% multi acid, and 1% urea. The ruminal fluid was then transported to the laboratory within 20 min from slaughter in a pre-warmed flask at 39 °C to maintain its integrity. Upon arrival at the laboratory, it was filtered through layers of cheesecloth and then mixed with the buffer solution at a ratio of 1:4 (*v*/*v*) [24] under continuous flushing with carbon dioxide (CO_2_) at 39 °C to mimic the ruminal environment [24]. 

The samples, prepared as described in Section 2.1, were divided into 4 Ankom Daisy jars. Each jar contained 9 bags of mixed hays (3 hays weighed in triplicate) and 2 L of prewarmed mixed buffer–ruminal solution. PET MPs were added to each jar at different concentrations: 0, 5, 10, and 15 g PET MPs/L buffer–ruminal solution, respectively, in jar 1, 2, 3, and 4. The jars were flushed with CO_2_ and placed in the Ankom Daisy ^II^ Incubator for 48 h at 39 °C, with a constant rotation at 1 rpm to simulate ruminal motility [25]. After the incubation period, the bags were washed in a turbine washing machine with cold water to remove any residue. They were then dried in a forced-air oven at 60 °C for 48 h to ensure that all moisture was removed. At the end of the drying process, the bags were weighed to determine the DM of mixed hay residue after ruminal incubation, according to the procedures of AOAC [18].

After determining the DM of mixed hay residues post-ruminal incubation, bags containing undegraded mixed hay from each jar (from step 1, ruminal phase) were used as follows: 3 bags to determine the CP of mixed hay and another 3 bags for NDF and ADF analysis. The CP of the mixed hay residues was measured using the Kjeldahl method, according to AOAC [18], while the NDF and ADF were determined using the Ankom 200 Fiber Analyzer (Ankom Technology, Macedon, NY, USA), following the procedures of Van Soest et al. [19].

*Step* *2.*  *Gastric digestion of mixed hay residue after ruminal incubation*

Gastric digestion was carried out by incubating the remaining 3 bags from each jar that underwent step 1 into 4 jars with 2 L of pre-warmed buffer solution at pH 1.9 (0.1 N HCl) with 1 g/L of pepsin (P-7000, Sigma, St. Louis, MO, USA) and the same doses of PET MPs (0, 5, 10, and 15 g PET MPs/L gastric solution). After 1 h of incubation in an Ankom Daisy^II^ Incubator, all the bags were washed under running tap water to remove any residual gastric contents.

*Step* *3.*  *Intestinal digestion of mixed hay residue after ruminal and gastric incubation*

Intestinal digestion was successively conducted after step 2. The bags used in step 2 were placed into 4 jars with 2 L of pre-warmed buffer solution at pH 7.75 (0.5 M KH_2_PO_4_) with 3 g/L of pancreatin (P-7545, Sigma, St. Louis, MO, USA), 50 ppm of thymol, and the same concentrations of PET MPs and incubated in an Ankom Daisy^II^ Incubator for 24 h.

After intestinal digestion, all bags were washed in a turbine washer to remove any remaining residue and dried in a forced-air oven at 60 °C for 48 h, to determine the DM of the mixed hay residue after ruminal–gastro-intestinal incubation according to the AOAC [18]. The CP of the mixed hay residue after ruminal–gastro-intestinal incubation was determined using the Kjeldahl method according to the AOAC [18].

### 2.4. In Vitro Ruminal Degradability and Gastro-Intestinal Digestibility Calculations

After all the analysis, the values of ruminal degradability, gastro-intestinal digestibility, and total tract digestibility were calculated as follows:

(2) $${DM}_{deg}=\frac{{DM}_{hay\;before\;ruminal\;incubation\;}-{DM}_{hay\;residue\;after\;ruminal\;incubation\;}}{{DM}_{hay\;before\;ruminal\;incubation\;}}\times 1000$$

(3) $${CP}_{deg}=\frac{{CP}_{hay\;before\;ruminal\;incubation\;}-{CP}_{hay\;residue\;after\;ruminal\;incubation\;}}{{CP}_{\;hay\;before\;ruminal\;incubation\;}}\times 1000$$

(4) $${NDF}_{deg}=\frac{{NDF}_{hay\;before\;ruminal\;incubation\;}-{NDF}_{hay\;residue\;after\;ruminal\;incubation}}{{NDF}_{\;hay\;before\;ruminal\;incubation\;}}\times 1000$$

(5) $${ADF}_{deg}=\frac{{ADF}_{hay\;before\;ruminal\;incubation\;}-{ADF}_{hay\;residue\;after\;ruminal\;incubation}}{{ADF}_{\;hay\;before\;ruminal\;incubation\;}}\times 1000$$

(6) $${DM}_{dig}=\frac{{DM}_{hay\;residue\;after\;ruminal\;incubation\;}-{DM}_{hay\;residue\;after\;ruminal-gastro-intestinal\;incubation}}{{DM}_{hay\;residues\;after\;ruminal\;incubation\;}}\times 1000$$

(7) $${CP}_{dig}=\frac{{CP}_{hay\;residue\;after\;ruminal\;incubation}-{CP}_{hay\;residue\;after\;ruminal\;-\;gastro\;-\;intestinal\;incubation}}{{CP}_{hay\;residue\;after\;ruminal\;incubation}}\times 1000$$

(8) $${DM}_{deg+dig}=\frac{{DM}_{hay\;before\;ruminal\;incubation}-{DM}_{hay\;residue\;after\;ruminal\;-\;gastro\;-\;intestinal\;incubation}}{{DM}_{hay\;before\;ruminal\;incubation}}\times 1000$$

(9) $${CP}_{deg+dig}=\frac{{CP}_{hay\;before\;ruminal\;incubation}-{CP}_{hay\;residue\;after\;ruminal\;-\;gastro\;-\;intestinal\;incubation}}{{CP}_{hay\;before\;ruminal\;incubation}}\times 1000$$

where

▪ DM_deg_ is the dry matter degradability of mixed hay in mg/g dry matter of mixed hay;
▪ CP_deg_ is the crude protein degradability of mixed hay in mg/g dry matter of mixed hay;
▪ NDF_deg_ is the neutral detergent fiber degradability of mixed hay in mg/g neutral detergent fiber of mixed hay;
▪ ADF_deg_ is the acid detergent fiber degradability of mixed hay in mg/g acid detergent fiber of mixed hay;
▪ DM_dig_ is the dry matter gastro-intestinal digestibility of ruminal non-degraded residue of dry matter of mixed hay in mg/g;
▪ CP_dig_ is the crude protein gastro-intestinal digestibility of ruminal non-degraded residue of crude protein in mg/g;
▪ DM_deg+dig_ is the dry matter total tract degradability and digestibility of mixed hay in mg/g dry matter of mixed hay;
▪ CP_deg+dig_ is the crude protein total tract degradability and digestibility of mixed hay in mg/g crude protein of mixed hay.

### 2.5. Statistical Analysis

All the data were statistically analyzed using a one-way analysis of variance (ANOVA) with PROC GLM of the SAS software (version 9.1; SAS Institute, Cary, NC, USA). The statistical significance of the differences between the mean values was assessed using the Tukey test. Orthogonal polynomial contrast was used to detect linear and quadratic effects in increasing PET MP levels. A significant difference was declared at a *p*-value < 0.05.

## 3. Results

### 3.1. Ruminal Degradability

The effects of PET MPs contamination on the DM_deg_, CP_deg_, NDF_deg_, and ADF_deg_ are summarized in Table 2. Overall, the increment in PET MPs level in the buffer–ruminal solution (at 0, 5, 10, and 15 g/L) linearly decreased (*p*-value < 0.05) the CP_deg_ and NDF_deg_ of the mixed hay. In particular, at the medium and high doses of PET MPs, the CP_deg_ declined by 9 and 16% respectively and the NDF_deg_ decreased by 9 and 13% respectively. However, the PET MPs did not influence the DM_deg_ and ADF_deg_ (*p*-value > 0.05) at any level.

### 3.2. Gastro-Intestinal Digestibility

The effects of PET MPs on the DM_dig_ and CP_dig_ are shown in Table 3. The contamination of the gastro-intestinal system with different doses of PET MPs at 0, 5, 10, and 15 g/L did not affect the DM_dig_ of the mixed hay (*p*-value > 0.05). However, it had a quadratic effect on the CP_dig_. In particulary it diminished the CP_dig_ at a low addition of PET MPs.

### 3.3. Total Tract Digestibility

The in vitro effects of PET MPs at 0, 5, 10, and 15 g/L of the buffer–ruminal and gastro-intestinal solutions on the DM_deg+dig_ and CP_deg+dig_ of the mixed hay is shown in Table 4. The DM _deg+dig_ of the mixed hay remained the same (*p*-value > 0.05) for all PET MP additions compared to the control. However, increasing the level of MPs from 0 to 5, 10, and 15 g/L of the buffer–ruminal and gastro-intestinal solutions led to a linear decrease in the CP_deg+dig_ of the mixed hay, with no significant differences between low, medium, and high levels of PET MPs.

## 4. Discussion

The effects of the presence of MPs in the ruminal–gastro-intestinal system on the digestive efficiency of ruminants have not yet received sufficient attention. Our in vitro study found that the addition of PET MPs to the ruminal–gastro-intestinal digestive system of bulls reduced their ability to degrade and digest certain mixed hay nutrient components, such as NDF and CP. However, this addition did not affect the degradation and digestion of the DM of the mixed hay. These results align with a previous in vivo study in sheep, which demonstrated that the daily intake of 100 mg of polystyrene MPs at a particle size of 50 µm reduced the apparent digestion of some dietary components, such as NDF and EE fraction, while the apparent digestion of the DM of diets remained unchanged [15]. This consistency could underscore a potential negative impact of MPs on the ruminant digestion’s ability to degrade some nutrient compounds of feeds. The results from our study are further supported by in vivo research on aquatic animals that proved the exposure of mussels to polyethylene MPs and polystyrene MPs caused significant changes in digestive enzyme activities [26]. These modifications in enzyme activities may explain the negative effects detected on specific compounds (CP and NDF) and maybe the increased degradation and digestion of other nutrient compounds not analyzed in our study. This selective effect could be due to changes in the microbial population in the digestive system, such as increased *Bacteroidetes* and *Actinobacteria* and decreased *Oriobacteriales incertae Sedis* and *Prevotellaceae YAB2003*, as proved in in vivo studies on the ruminal microbiota of sheep contaminated by polyester MPs [15], the intestinal microbiota of mice contaminated by PET MPs [11], and the intestinal microbiota of zebrafish contaminated by PET MPs [27] and an in vitro study on a human simulated digestive system contaminated by polyethylene MPs [28].

In the ruminal phase, PET MPs reduced the NDF_deg_ with significant differences between levels of 0 and 10, and 15 g/L, with reductions at medium and high doses by 9% and 13%, respectively., while it did not alter the ADF_deg_. This selective inhibition of fiber compounds aligns with studies on aquatic animals. These indicate that mussels exposed to polyethylene MPs and polystyrene MPs showed a reduction in xylanase activity, crucial for degrading the hemicellulose part of NDF, but not in cellulase activity, crucial for degrading the cellulose part of ADF and NDF. Therefore, ADF degradation remains unaffected [26]. This reduction in the NDF_deg_ can lead to lower feed intake and diminish ruminal performance, resulting in significant economic losses for producers [29]. In addition, in this phase, PET MPs also reduced the CP_deg_ with significant differences between levels of 0 and 10, and 15 g/L, with reductions at medium and high doses by 9% and 16% respectively. This reduction can be attributed to the potential inhibition of protease enzyme activity produced by the ruminal microbiota, as proved in a previous study in mussels exposed to polystyrene MPs [30]. This study [30] showed that protease activity in the digestive system of these aquatic animals decreased with increasing polystyrene MP concentrations. These findings indicate a broader impact of MPs on protein degradation in both aquatic animals and ruminants. The reduction in the CP_deg_ and NDF_deg_ of the mixed hay, detected only at a medium and high addition of PET MPs, can be attributed to the ability of the ruminal microbiota to release terephthalic acid from PET MPs [10], which can be toxic at high concentrations [31]. Additionally, PET MPs can interact with the gut microbiota and impair its normal function [11,13,27]. However, this interaction may be more pronounced at higher levels of PET MPs, where a greater quantity of particles can more significantly disrupt the balance and activity of microbial populations.

In the gastro-intestinal phase, the CP_dig,_ was significantly reduced by the presence of PET MPs at a low dose, decreasing by 8%. This negative effect, observed only in the gastro-intestinal phase at this dose and not in the ruminal phase, could be due to gastric acid altering the characteristics of PET MP particles or changing their shape, potentially making them more bioactive [22]. Alternatively, the MPs might absorb enzymes, like pepsin, thereby reducing their efficacy [14,30] or they could reduce the activity of pancreatic enzymes [32]. However, at medium and high levels of PET MP contamination, the CP_dig_ of mixed hay did not show significant differences in comparison to the control. This unexpected result suggests that the relationship between PET MP concentrations and their impact on the CP_dig_ may not be linear and could be influenced by other factors. Future studies should aim to elucidate these mechanisms, to better understand the complex interactions between PET MPs and the CP_dig_ in the gastro-intestinal tract.

In addition, PET MPs significantly decreased the CP_deg+dig_ of the mixed hay at all doses. Similar to our finding, previous in vivo studies on lambs showed that daily ingestion of 100 mg of polystyrene MPs at 100 μm reduced the apparent CP digestion [15]. This reduction in the CP_deg+dig_ can decrease feed efficiency and ruminant productivity, increase feed costs, and ultimately reduce overall farm profitability and sustainability [33]. Additionally, the decreased CP_deg+dig_ contributes to increased nitrogen residue in manure, which is converted into ammonia and nitrous oxide that volatilize into the atmosphere, contributing to air pollution and greenhouse gas emissions [33,34].

## 5. Conclusions

This study has demonstrated, for the first time, the adverse effects of PET MPs pollution on the digestive activity of the ruminal–gastro-intestinal system of ruminants. Significant adverse effects were observed at PET MP concentrations of 10 and 15 g/L in the buffer–ruminal solution. Increasing concentrations of PET MPs in the ruminant digestive system led to a linear decline in the ability to degrade the NDF content of mixed hay. Furthermore, the addition of PET MPs impaired the performance of the ruminal–gastro-intestinal digestive system on the CP_deg+dig_ of mixed hay, across all levels of addition, albeit not uniformly throughout the different digestive phases. Specifically, PET MPs negatively affected the CP_deg_ during the ruminal phase at medium and high concentrations, whereas their detrimental effects on the CP_dig_ during the gastro-intestinal phase were observed only at the lowest concentrations. Further research is needed to explore these mechanisms and clarify the specific factors contributing to the detrimental effects of PET MPs on ruminant digestion. Moreover, it should be assessed whether the observed effects are attributable to the chemical properties of the MP material itself, additives in the MPs, degradation products of the MP material, residual starting materials from the polymerization process, or a combination of these factors. Future studies can build upon these initial findings by evaluating the effects of additional doses of PET MPs.

## Figures and Tables

**Table 1:** Chemical composition of mixed hays collected in three farms (mg/g dry matter).

Chemical Composition	Farm 1	Farm 2	Farm 3
Dry matter ^1^	882.8	881.6	903.8
Ash	97.4	99.8	112.7
Ether extract	16.5	17.3	17.5
Crude protein	107.1	105.5	110.5
Neutral detergent fiber	718.8	697.1	684.4
Acid detergent fiber	386.1	375.5	370.3
Acid detergent lignin	60.6	55.1	54.1
Non fiber carbohydrates	60.2	80.3	74.9

^1^ Dry matter in mg/g fresh matter.

**Table 2:** Effects of polyethylene terephthalate microplastics in buffer–ruminal solutions on ruminal degradability of nutrient compounds of mixed hay (mg/g).

	PET MPs Added in Buffer–Ruminal Solutions (g/L)				SEM	*p*-Value	Orthogonal Polynomial Contrast	
	0	5	10	15			Linear	Quadratic
DM_deg_	504	486	488	492	38.9	0.749	0.353	0.212
CP_deg_	446 ^a^	417 ^ab^	408 ^bc^	375 ^c^	34.4	0.003	0.0003	0.888
NDF_deg_	469 ^a^	446 ^ab^	426 ^b^	409 ^b^	31.9	0.003	0.0002	0.790
ADF_deg_	375	360	348	347	66.7	0.760	0.309	0.711

^a–c^ Values within row with different superscripts differ significantly at *p*-value < 0.05; ADF_deg_: acid detergent fiber degradability of mixed hay; CP_deg_: crude protein degradability of mixed hay; DM_deg_: dry matter degradability of mixed hay; PET MPs: polyethylene terephthalate microplastics; NDF_deg_: neutral detergent fiber degradability of mixed hay; SEM: standard errors of means.

**Table 3:** Effects of addition of polyethylene terephthalate microplastics to gastro-intestinal solutions on gastro-intestinal digestibility of ruminal undegraded dry matter and crude protein content of mixed hay (mg/g).

	PET MPs Added in Gastro-Intestinal Solutions (g/L)				SEM	*p*-Value	Orthogonal Polynomial Contrast	
	0	5	10	15			Linear	Quadratic
DM_dig_	189	204	229	226	42.1	0.193	0.054	0.550
CP_dig_	553 ^a^	506 ^b^	520 ^ab^	538 ^ab^	34.1	0.04	0.482	0.011

^a–c^ Values within row with different superscripts differ significantly at *p*-value < 0.05; CP_dig_: crude protein gastro-intestinal digestibility of ruminal non-degraded residue of crude protein of mixed hay; DM_dig_: dry matter gastro-intestinal digestibility of ruminal non-degraded residue of dry matter of mixed hay; PET MPs: polyethylene terephthalate microplastics; SEM: standard errors of the means.

**Table 4:** Effects of addition of polyethylene terephthalate microplastics to buffer–ruminal gastro-intestinal solutions on total tract digestibility of dry matter and crud protein content of mixed hay (mg/g).

	PET MPs Added in Buffer–Ruminal Gastro-Intestinal Solutions (g/L)				SEM	*p*-Value	Orthogonal Polynomial Contrast	
	0	5	10	15			Linear	Quadratic
DM_deg+dig_	600	590	606	607	33.8	0.61	0.239	0.885
CP_deg+dig_	752 ^a^	712 ^b^	716 ^b^	712 ^b^	22.0	0.001	0.001	0.021

^a,b^ Values within row with different superscripts differ significantly at *p*-value < 0.05; CP: crude protein tract degradability and digestibility of mixed hay; DM: dry matter total tract degradability and digestibility of mixed hay; MPs: polyethylene terephthalate microplastics; SEM: standard errors of means.

## Data Availability

The data presented in this study are available on request from the corresponding author.
